# Interpolation and Imputation Strategies for Missing Segments in Continuous Pressure-Flow Cerebral Bio-Signals: A Systematic Scoping Review

**DOI:** 10.3390/s26103134

**Published:** 2026-05-15

**Authors:** Isuru Sachitha Herath, Izabella Marquez, Julia Ryznar, Xue Nemoga-Stout, Yushu Shao, Rakibul Hasan, Amanjyot Singh Sainbhi, Kevin Y. Stein, Nuray Vakitbilir, Noah Silvaggio, Mansoor Hayat, Jaewoong Moon, Tobias Bergmann, Frederick A. Zeiler

**Affiliations:** 1 Department of Biomedical Engineering, Price Faculty of Engineering, University of Manitoba, Winnipeg, MB R3T 5V6, Canada; hasanr2@myumanitoba.ca (R.H.); amanjyot.s.sainbhi@gmail.com (A.S.S.); steink34@myumanitoba.ca (K.Y.S.); vakitbir@myumanitoba.ca (N.V.); bergmant@myumanitoba.ca (T.B.); frederick.zeiler@umanitoba.ca (F.A.Z.); 2 Undergraduate Engineering, Price Faculty of Engineering, University of Manitoba, Winnipeg, MB R3T 5V6, Canada; marquezi@myumanitoba.ca (I.M.); ryznarj@myumanitoba.ca (J.R.); nemogasx@myumanitoba.ca (X.N.-S.); 3 Department of Statistics, Faculty of Science, University of Manitoba, Winnipeg, MB R3T 2M8, Canada; shaoy4@myumanitoba.ca; 4 Department of Human Anatomy and Cell Science, Rady Faculty of Health Sciences, University of Manitoba, Winnipeg, MB R3E 0J9, Canada; silvaggn@myumanitoba.ca; 5 Section of Neurosurgery, Department of Surgery, Rady Faculty of Health Sciences, University of Manitoba, Winnipeg, MB R3A 1R9, Canada; mansoor.hayat@umanitoba.ca (M.H.); jaewoong.moon@umanitoba.ca (J.M.); 6 Department of Clinical Neuroscience, Karolinska Institutet, 171 77 Stockholm, Sweden; 7 Division of Anaesthesia, Department of Medicine, Addenbrooke’s Hospital, University of Cambridge, Cambridge CB2 0QQ, UK; 8 Pan Clinic Foundation, Winnipeg, MB R3M 3E4, Canada

**Keywords:** continuous pressure-flow cerebral bio-signals, interpolation, imputation, missing data, neurocritical care monitoring

## Abstract

**Objective**: Continuous pressure-flow cerebral bio-signals are critical for monitoring cerebrovascular dynamics but are often disrupted by missing data segments caused by artifacts from a variety of sources. These missing segments refer to segments of the signal that do not contain any valid physiological data. Such interruptions fragment the signals, resulting in discontinuities that compromise their overall integrity. Therefore, reconstructing missing values and preserving signal continuity are essential for ensuring the stable computation of signal trajectories and the accuracy of derived cerebrovascular indices. **Methods**: To address this issue, this systematic scoping review aimed to identify and synthesize existing interpolation and imputation strategies for handling missing segments in continuous pressure-flow cerebral bio-signals. Following the Cochrane and Preferred Reporting Items for Systematic Reviews and Meta-Analysis guidelines, a comprehensive search of five electronic databases was conducted from their inception to 24 September 2024, and updated on 16 June 2025, using a detailed search string. **Results**: The initial searches yielded 19,403 results, and 8 studies were filtered and included in the review. All included studies employed interpolation techniques, such as linear and spline interpolation algorithms, to correct distorted signal segments. However, none of the included studies directly utilized interpolation or imputation strategies to reconstruct or completely fill missing data segments. **Conclusions**: This reveals a critical knowledge gap, as no study has systematically addressed the utilization of interpolation or imputation strategies for missing segments in pressure-flow cerebral bio-signals. Therefore, this systematic review emphasizes the need for specialized methodologies and standardized frameworks to enable reliable recovery of missing data segments in pressure-flow cerebral bio-signals, which is critical for advancing real-time neurocritical care monitoring and experimental neuroscience/psychological research. **Significance**: This systematic review lays the groundwork for future research into physiologically informed interpolation and imputation strategies for pressure-flow cerebral bio-signals in clinical and research applications.

## 1. Introduction

Cerebral pressure-flow bio-signals are time-varying physiological signals that capture the complex dynamic relationships of intracranial pressure, cerebral blood flow, and cerebral oxygenation within the brain [1,2]. Intracranial pressure (ICP), cerebral blood flow (CBF), cerebral blood flow velocity (CBFV), and cerebral oxygenation-related measures such as oxyhemoglobin (HbO_2_), deoxyhemoglobin (HHb), and regional oxygen saturation (rSO_2_) are commonly monitored pressure-flow cerebral bio-signals used in both clinical and research settings to characterize dynamic cerebrovascular function in humans and large mammals [3,4,5].

Acquisition of continuous pressure-flow cerebral bio-signals is accomplished using both invasive and non-invasive cerebral monitoring devices. High-frequency time series of ICP signals are obtained using invasive probes placed in the cranial cavity [6,7]. In contrast, CBFV and cerebral oxygenation-related measures (i.e., HbO_2_, HHb, and rSO_2_) are obtained non-invasively using transcranial Doppler ultrasound (TCD) and near-infrared spectroscopy (NIRS), respectively [8,9,10]. Furthermore, functional near-infrared spectroscopy (fNIRS), a specialized application of NIRS, utilizes cerebral HbO_2_ and HHb to map and classify brain activities [11,12]. Collectively, these monitoring techniques provide continuous, high-resolution pressure-flow cerebral bio-signals that are crucial for characterizing cerebrovascular dynamics and assessing cerebrovascular functions, oxygen delivery, and nutrient delivery mechanisms. Clinical applications of evaluating and assessing cerebrovascular functions and mechanisms by utilizing cerebral signals include forecasting of future physiologic events/insults [13,14], and derivation of various cerebral autoregulatory indices continuously in real-time, which can be leveraged to guide continuous individualized therapeutic strategies for patients in neurocritical care [15,16,17,18,19,20]. Beyond clinical applications, cerebral pressure-flow signals, such as NIRS, are widely used in experimental neuroscience and psychological research to investigate neurovascular coupling [21,22] and task or stimulus-evoked changes [23,24,25] in cerebral hemodynamics.

Although pressure-flow cerebral bio-signals acquired through cerebral monitoring devices can be leveraged to derive real-time continuous physiological metrics, they share common pitfalls inherent to sensor-based measurements [26,27]. Pertaining to sensor readings, these signals are vulnerable to various sources of degradation and contamination, including artifacts, transient device disruptions, loss of signal quality, and gradual signal drifts. These factors can result in missing data segments or portions of the recording that are unusable due to compromised reliability, ultimately fragmenting what should be a continuous, high-quality signal stream into discontinuous intervals, containing gaps and corrupted segments.

In the context of sensor measurements, missing values represent the complete absence of valid data when the sensor failed to acquire any pressure-flow cerebral bio-signal at specific time points. Such time points in the pressure-flow cerebral bio-signal are often caused by brief sensor disconnections, signal dropout during patient movement, or temporary device malfunction [28,29], resulting in intervals with null or zero entries. In addition, sensor readings can be contaminated by artifacts that distort or degrade signal quality. These artifacts arise due to calibration drift, excessive motion, poor probe coupling in non-invasive sensors, electromagnetic or optical interference, or physiological instabilities such as hemodynamic fluctuations and arrhythmias [30,31].

In some scenarios, excessive artifact contamination can corrupt signal segments so severely that no valid physiological information can be extracted, effectively causing them to function as missing segments despite the presence of recorded pressure-flow cerebral bio-signals. These extreme artifact-induced segments differ from typical artifactual intervals, which contain physiologically informed data that can often be recovered through appropriate filtering or artifact-removal algorithms, allowing restoration of the underlying physiological signal.

Both of these missing value intervals and extremely corrupted signal intervals share the characteristic of unreliable or absent physiological information, creating void gaps in the continuous data stream of pressure-flow cerebral bio-signals. To maintain consistency in terminology throughout this review, both types of data absences are collectively referred to as “missing segments” in continuous pressure-flow cerebral bio-signals. To define this concept formally, let *x*(*t*) denote a continuous pressure-flow cerebral bio-signal defined for $t\in \left[0,\;T\right]$, where *T* > 0 is the recording duration. A missing segment is then defined as a closed time interval $\left[t_{a},\;t_{b}\right]\subset \left[0,\;T\right]$, with $t_{a}<t_{b}$, over which no reliable physiological values are present, such that

$$x\left(t\right)=\left\{\begin{array}{cc} x_{obs}\left(t\right), & t<t_{a}\;or\;t>t_{b},\\ \;missing, & t_{a}\leq t\leq t_{b}. \end{array}\right.$$

where *x_obs_*(*t*) denotes the observed pressure-flow cerebral bio-signal, and “missing” indicates complete absence or unreliability of physiological information.

Discontinuities caused by missing segments in pressure-flow cerebral bio-signals significantly reduce the reliability, integrity, and statistical power of the data for deriving continuous physiological metrics [32,33]. In most research contexts, where uninterrupted time series are not critical, such missing and corrupted intervals can often be discarded during preprocessing [34,35,36,37]. However, in certain research settings where continuous pressure-flow cerebral bio-signals are essential or, more crucially, at the bedside in clinical environments where these pressure-flow cerebral bio-signals provide real-time assessments of cerebral autoregulatory dynamics, simply discarding missing or unusable segments is not a viable option. At the point of care, clinical teams depend on uninterrupted high-resolution signal streams to monitor cerebrovascular dynamics and guide individualized therapeutic strategies [38,39,40,41]. In these settings, every segment of valid data is scarce and valuable, and preserving continuity is critical to ensure stable computation of signal trajectories into the future or for deriving more complex cerebrovascular physiologic metrics. Thus, missing and unusable signal segments must be filled with the most plausible physiological values that approximate what the pressure-flow cerebral bio-signal would have been at those time segments.

**Figure 1:**
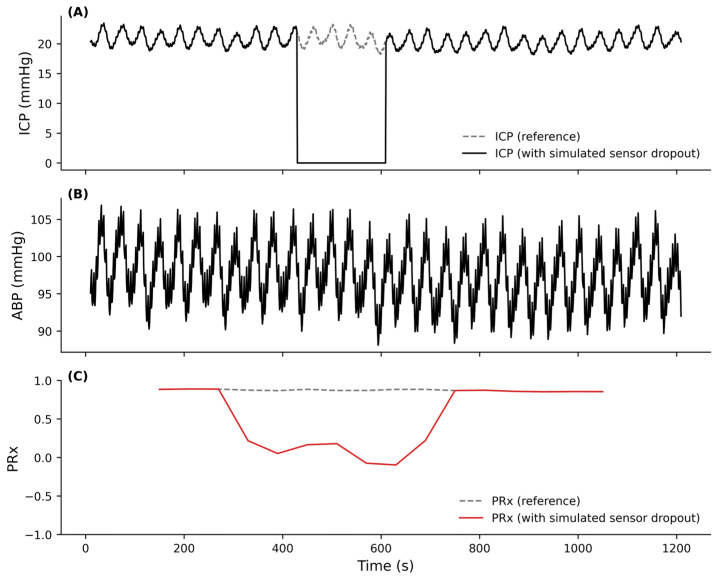
Impact of intracranial pressure (ICP) signal dropout on real-time pressure reactivity index (PRx) computation. Missing data segments in the ICP signal propagate through the PRx computation and introduce distortion in the index trajectory. (**A**) ICP signal from the PhysioNet CHARIS database [42,43] with a simulated sensor dropout illustrating a missing data segment. (**B**) Arterial blood pressure (ABP) signal used for PRx computation. (**C**) PRx computed using a 5 min moving window with 1 min updates, showing divergence between the trajectory derived from the ICP signal with a missing segment and the reference trajectory derived from the intact ICP signal.

For example, during invasive ICP monitoring of a patient with traumatic brain injury (TBI), ICP sensor malfunction can cause periods where the ICP signal is lost, creating missing gaps in the real-time computation of a cerebrovascular reactivity index, such as the pressure reactivity index (PRx). These missing ICP signal segments introduce distortions/discontinuities in the trajectories of these cerebrovascular indices (as depicted in Figure 1), thereby distorting the clinical decision-making process.

In general, to address missing segments in continuous signals and time series, a wide range of methodologies have been developed to reconstruct or estimate missing segments and restore data continuity. Traditional approaches, such as interpolation-based methods (including linear, spline, and polynomial interpolation) [44,45,46] and statistical imputation techniques [46], are widely employed due to their simplicity and computational efficiency. Here, we use interpolation to refer to methods that reconstruct missing points within a time series by exploiting the local temporal continuity of the same signal (for example, estimating a short missing gap in an ICP waveform by fitting a spline between the last valid sample before the gap and the first valid sample after it), whereas imputation denotes broader statistical or model-based procedures that can use information beyond the immediate neighborhood to fill in missing data (such as filling missing gaps with local mean values of the signal) [47,48]. Apart from these traditional approaches, model-based estimation frameworks, such as autoregressive moving average (ARMA) models and related time-series techniques, have also been utilized to impute missing segments [49]. In recent years, machine learning and deep learning methods such as Recurrent Neural Networks (RNNs), Long Short-Term Memory (LSTM) networks, Generative Adversarial Networks (GANs), and Transformer architectures have emerged as powerful techniques for reconstructing missing signal segments, with the ability to model complex non-linear relationships and capture long-range temporal dependencies [50,51].

Despite the existing methodologies and research on interpolation and imputation strategies for missing segments in general continuous time series signals, there remains a limited understanding of how these methods have been applied and validated in the context of pressure-flow cerebral bio-signals. Only a few studies have evaluated and researched this specific application, and those have primarily been based on conventional interpolation methods. Therefore, to address this knowledge gap, the objective of the present systematic scoping review is to comprehensively identify, explore, and synthesize existing literature on interpolation and imputation strategies for reconstructing missing segments in continuous pressure-flow cerebral bio-signals, both qualitatively and, where possible, quantitatively. This systematic scoping review aims to provide foundational insights to guide researchers in developing more effective and domain-specific interpolation and imputation approaches for continuous pressure-flow cerebral bio-signals.

## 2. Methodology

This systematic scoping review was conducted according to the methodology outlined in the Cochrane Handbook for Systematic Reviews [52]. The reporting of the results was done by adhering to the guidelines outlined in the Preferred Reporting Items for Systematic Reviews and Meta-Analysis (PRISMA) guidelines, with the PRISMA Extension for Scoping Review [53,54]. The review objectives for the search strategy were collaboratively developed by FAZ, IM, TB, and ISH. Assistance with the article filtering process was provided by TB, IM, JR, and XNS. The completed PRISMA checklist can be found in Supplementary File S1.

### 2.1. Ethical Considerations

All the articles included in this systematic scoping review were previously published in peer-reviewed journals. Therefore, no additional ethical approval is required for this systematic scoping review.

### 2.2. Search Question and Inclusion/Exclusion Criteria

The primary search question investigated in this systematic scoping review was “What strategies have been developed for interpolating or imputing missing segments in continuous pressure-flow cerebral bio-signals?” Secondary questions included: examining the effectiveness of interpolation and imputation strategies; identifying which specific interpolation/imputation techniques have been developed for particular types of pressure-flow cerebral bio-signals; evaluating the impact of interpolation/imputation on temporal dynamics, inter-signal relationships, and subject-level outcomes; characterizing the types of interpolation and imputation algorithms proposed and applied; assessing their feasibility for real-time implementation; and evaluating how the effectiveness of these interpolation and imputation strategies was measured.

For this review, continuous pressure-flow cerebral bio-signals were defined as time-varying physiological parameters, such as ICP, CBF, CBFV, HHb, HbO_2_, and rSO_2_, which were obtained either invasively or non-invasively at a minimum sampling frequency of 0.1 Hz or higher. A minimum sampling frequency was specified to ensure that the data represent continuous signals capable of capturing dynamic fluctuations in cerebrovascular physiology over time in the included studies. A threshold of 0.1 Hz was chosen as a conservative lower bound because it provides sufficient temporal resolution to resolve slow, physiologically relevant pressure-flow oscillations, while excluding sparsely sampled measurements [55,56,57].

This review included full-text, peer-reviewed manuscripts published in English that examined interpolation or imputation strategies applied to continuous pressure-flow cerebral bio-signals acquired from human or animal subjects. Non-English publications, abstracts, and studies where interpolation or imputation methods were inseparably combined with artifact correction techniques, preventing independent evaluation of interpolation/imputation performance, were excluded. Studies solely based on simulated/synthesized continuous pressure-flow cerebral bio-signal datasets were also excluded, as simulated/synthesized signals do not fully reflect true underlying cerebral physiological dynamics. This is because simulated data often simplify cerebrovascular regulation, omit clinically relevant sources of variability, and may not capture the morphology and temporal coupling of true pressure-flow cerebral bio-signals [58]. Thus, for this review, we only included studies that tested and validated interpolation and imputation strategies on real continuous pressure-flow cerebral bio-signal datasets, including real signals with artificially induced missing segments or artifacts, to ensure that their performance and limitations are assessed under realistic clinical conditions and to provide a more robust foundation for future translation to bedside applications.

### 2.3. Search Strategy

Initially, a systematic literature search was conducted across five electronic databases: BIOSIS, SCOPUS, EMBASE, PubMed, and the Cochrane Library, covering all available literature from each database’s inception to 24 September 2024. A dedicated search string was formulated for all databases, incorporating a combination of keywords related to interpolation and imputation strategies for missing segments in all cerebral bio-signals. The detailed search string for all the databases is provided in Supplementary File S2. The search results from all databases were combined into a unified dataset while precisely removing duplicate entries. The de-duplicated results were then filtered to identify studies investigating pressure-flow cerebral bio-signals, distinguishing them from studies on other cerebral bio-signals. The search was then updated on 16 June 2025, using the same search string for the same five databases.

### 2.4. Study Selection

A two-stage, two-reviewer approach was utilized to manually review all studies extracted from the initial literature search. At the first stage, two reviewers (TB and IM) independently assessed the eligibility of studies by screening titles and abstracts using the predefined inclusion and exclusion criteria. Studies that met the criteria at this stage were then carried forward to full-text screening. At the second stage, the same two reviewers (TB and IM) independently evaluated the full-length manuscripts of each study. Any disagreements between the two reviewers at either stage were resolved by an independent third reviewer (FAZ). This independent dual-reviewer process with third-party adjudication was used to minimize individual reviewer bias, ensure consistent application of the screening criteria, and reduce the risk of selection bias.

Initially, the search strategy for this systematic scoping review was designed to evaluate interpolation and imputation strategies for missing segments in all cerebral bio-signals in the current literature. However, given the large volume of results, the heterogeneous nature of cerebral bio-signals, and to maintain a clear focus in this scoping review, the search results were separated based on cerebral bio-signal type into pressure-flow cerebral physiological signals and cerebral electrophysiological signals. Consequently, during the full-text evaluation in stage two, studies that solely investigated interpolation or imputation strategies for missing segments in pressure-flow cerebral bio-signals were selected, while studies that focused solely on cerebral electrophysiological signals were excluded on the pretext of “wrong signal type”. This approach allowed the initial broad search to remain exhaustive at the cerebral bio-signal level, while the a priori separation into cerebral electrophysiological and cerebral pressure-flow branches limited selection bias at the level of signal type.

In study selection, studies that utilized real continuous pressure-flow cerebral bio-signals with naturally occurring artifacts or missing segments were included, along with studies that used real signals with simulated artifacts or simulated/induced missing segments. In contrast, studies that utilized simulated or synthesized continuous pressure-flow cerebral bio-signals were excluded, regardless of whether naturally occurring artifacts or missing segments had been incorporated into those data.

### 2.5. Data Collection

Key characteristics were collected from each study, including population characteristics, data or signal characteristics, and methodological information regarding interpolation and imputation approaches. Population characteristics comprised sample size, age, inclusion and exclusion criteria, and the participants’ clinical conditions or pathologies. Data or signal characteristics included the signal type, sensor or instrument used, anatomical recording location, sampling rate, duration of acquisition, and any simultaneously recorded bio-signals. If simulated data or intentionally introduced artifacts/missing values were used in a study, these scenarios were also recorded. Information regarding the theoretical foundation of the proposed interpolation/imputation methods in each study was extracted, along with their effectiveness and computational complexity. The main statistical results of each study were also recorded. Analysis of the proposed interpolation and imputation strategy involved comparatively evaluating the effectiveness and computational complexity with other methods presented in the study. Limitations of each study were extracted as reported by the authors, and as identified through an independent critical evaluation conducted by the reviewer (ISH). These collected key characteristics were utilized for the analysis in this review and are presented in the Supplementary File S3 in a structured tabular form. Information extracted from each study (when available) was organized in the table under the headings described below.

1. Reference—author name, year, and citation.
2. Subject information—number of subjects, relevant clinical conditions or pathologies, any inclusion/exclusion criteria, and dataset descriptions.
3. Signal type, sampling rate, and measurements—type of cerebral pressure-flow bio-signal (e.g., ICP, CBFV, HbO_2_, HHb, rSO_2_), sampling rate, recording duration, and any additional measured variables.
4. Missing data characteristics and the detection technique used—how missing or corrupted segments were defined, induced, or identified, including gap length/pattern and the algorithm or criteria used for detection.
5. Sensor location—number of sensors and anatomical recording sites (e.g., middle cerebral artery, prefrontal cortex, motor cortex).
6. Interpolation technique used—detailed description of the interpolation/imputation method(s) applied to missing segments (e.g., linear, spline, model-based, machine learning) and computational hardware specification.
7. Methods compared—any baseline or comparison methods evaluated alongside the primary interpolation/imputation approach.
8. Evaluating the effectiveness of interpolation methods—quantitative and qualitative evaluation metrics used (e.g., RMSE, MAE, correlation, coherence, SNR) and how performance was assessed.
9. Study results and conclusion—main findings related to interpolation/imputation performance and the authors’ overall conclusions.
10. Limitations—limitations reported by the study authors and any additional constraints identified during this review.

### 2.6. Assessment of Bias

Since all the included studies in this review were published in peer-reviewed journals, biases associated with these studies were assumed to have been screened through the independent review process of the respective journals in which they were published. However, no formal bias assessment was conducted as part of this systematic scoping review.

## 3. Results

### 3.1. General Study Characteristics

**Figure 2:**
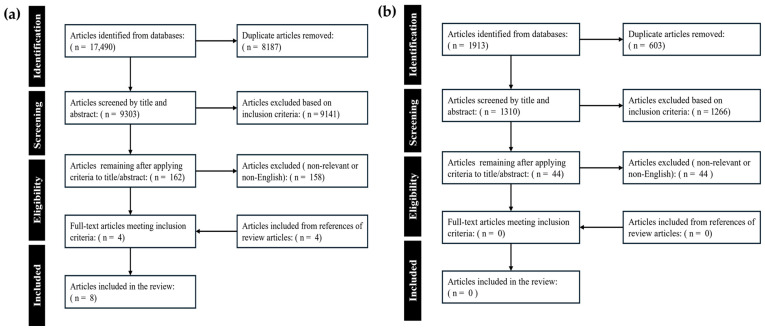
PRISMA flowchart for a systematically conducted scoping review from searches on (**a**) 24 September 2024, and updated on (**b**) 16 June 2025.

The results of this systematic scoping review were obtained through searching unique articles across five electronic databases (BIOSIS, SCOPUS, EMBASE, PubMed, and Cochrane Library) and the reference sections of each article based on the inclusion/exclusion criteria. The initial search yielded 17,490 articles. After filtering for duplicates, 9303 unique articles were reviewed in the first screening phase. From the first screening phase, 162 articles were deemed eligible based on the inclusion and exclusion criteria. In the second screening phase of full-text review, four articles were deemed eligible, and four additional articles were identified by screening through the reference sections of these articles. The updated search conducted on 16 June 2025 yielded 1913 articles, and after duplicate removal, 1310 unique articles were included and screened in the two-stage review. In the first screening phase, 44 studies were deemed eligible, but after full-text review, none were found to be eligible. The initial and updated searches yielded a total of 8 eligible studies based on inclusion and exclusion criteria. The screening process and selection outcomes are illustrated in the PRISMA flow diagrams in Figure 2a,b.

All 8 included studies were human-based studies, and no animal-based studies met the inclusion criteria. All 8 studies utilized interpolation strategies applied to pressure-flow cerebral bio-signals, and none employed imputation approaches for missing segments in these signals. Six studies examined pressure-flow cerebral bio-signals acquired from healthy subjects [35,59,60,61,62,63]. One study included both healthy controls and patients with acute stroke [64], while another did not explicitly report participants’ health status but was reasonably presumed healthy based on the study design [65]. The population demographics of the included studies comprised children (group mean age = 5.95 ± 2.96 years) [62,63] and adults (age ≥ 18 years) [35,60,61,64], while two studies [59,65] did not provide explicit information on age demographics.

Cerebral oxygenation measures and CBFV were the two main types of pressure-flow cerebral bio-signals examined for interpolation in the included studies. Seven of eight included articles focused on cerebral oxygenation measures derived from NIRS and fNIRS devices, with sampling rates ranging from 7.8 Hz to 50 Hz. NIRS and fNIRS sensors were placed in various cerebral regions, including the prefrontal areas [35,59,60], the motor cortex [65], both frontal and pre-motor cortices [61], bilateral frontal, temporal, and parietal cortices [62], and the left inferior frontal gyri [63]. Only one study [64] investigated CBFV by measuring cerebral blood flow velocity using TCD in the middle cerebral arteries at a sampling rate of 0.2 Hz.

In the included studies, missing or contaminated segments within pressure-flow cerebral bio-signals were identified either manually or through automated detection algorithms. Following identification, these affected segments were interpolated using various strategies.

The computational hardware specifications of the utilized interpolation strategies were largely underreported. Only one out of the eight included studies explicitly described the computational hardware used to evaluate its interpolation approach (a 3.4-GHz CPU running Windows 7, with no RAM specification provided) [60] and reported corresponding processing time, whereas none of the remaining studies reported either the hardware configuration or quantitative processing-time/run-time metrics for their interpolation approaches on pressure-flow cerebral bio-signals.

As the main objective of this systematic scoping review was to compile existing interpolation and imputation strategies applied to missing segments in continuous pressure-flow cerebral bio-signals, and because no eligible studies on imputation approaches for these signals were identified, findings were categorized into three interpolation-focused groups: (1) studies that employed standalone interpolation approaches, (2) studies that employed combined approaches which integrated interpolation with artifact correction or signal reconstruction techniques, and (3) comparative studies. For the studies with multi-layered approaches, the analysis focused specifically on evaluating the interpolation components within these pipelines. A comprehensive synthesis and critical evaluation of the identified interpolation techniques are presented in the subsequent sections. Extensive summaries of the included studies, organized according to the three classification categories, are provided in Supplementary Tables S2–S4 of Supplementary File S3.

### 3.2. Standalone Interpolation Approaches

Three studies were presented on the utilization of interpolation strategies for missing or contaminated segments in pressure-flow cerebral bio-signals [59,64,65], and these three studies utilized completely different interpolation approaches. The studies by Scholkmann et al. and Hayashi et al. employed spline interpolation [65] and possibilistic membership-based interpolation on NIRS pressure-flow cerebral bio-signals [59], respectively, while Eames et al. utilized linear interpolation on CBFV [64]. From these studies, summarized information regarding the subjects, signal types, and measurements, missing data characteristics and detection techniques used, sensor location, and interpolation strategy utilized are presented in Table 1.

In Scholkmann et al., cubic spline interpolation was employed in the proposed “Movement Artifact Reduction Algorithm (MARA)”, which first detected motion artifact segments in the signal by moving the standard deviation with a user-specified threshold [65]. MARA assumed that the motion artifact-contaminated segment contained both the underlying physiological signal and the artifact component. Then, cubic spline interpolation was applied to the motion artifact segment to fit a spline that represents the artifact behavior, which was subsequently subtracted from the contaminated segment, thereby recovering the artifact-removed segment. For the validation of the proposed MARA approach, three real NIRS datasets were simulated with artifact types: short impulses, base shifts, and temporally limited low-frequency oscillations, and corrected using the proposed MARA approach. Then, corrected NIRS datasets (with MARA) and corrupted NIRS datasets (without MARA) were compared against the original NIRS dataset with evaluation metrics of percent changes in Root Mean Square Error (RMSE), Percent Root Mean Squared Difference (PRD), Pearson correlation coefficient (*r*), and signal to noise ratio (SNR). These metrics demonstrated that the average percent changes in PRD, RMSE, and R were 89.7% decrease, 89.8% decrease, and 61.6% increase, respectively, in corrected NIRS datasets (with MARA) compared to the corrupted NIRS dataset (without MARA).

Hayashi et al. proposed a Possibilistic Data Interpolation-Bagging (pdi-Bagging) algorithm, which added virtual training data generated by possibilistic membership-based interpolation for misclassified data instances during brain activity classification model training [59]. This approach demonstrated an average final recognition rate of 93.33% for classifying brain activity (active vs. steady state) using NIRS data, which was higher than those of AdaBoost (92.99%), MultiBoost (92.30%), and REPTree (92.31%) models. Although this interpolation strategy was used with pressure-flow cerebral bio-signals, it is only applicable for developing supervised classification models and cannot be utilized to interpolate missing segments of signals in the time domain.

Linear interpolation was used to remove narrow spikes caused by ectopic heartbeats on the CBFV signals in the study by Eames et al. [64] and the effectiveness of interpolation was evaluated by the coherence value between CBFV and non-invasive blood pressure (NIBP) [64]. Although the authors noted that removal of ectopic heartbeats by linear interpolation significantly lowered coherence, it may have introduced noise and removed useful information from the CBFV signal.

As these included studies employed different interpolation approaches with different types of pressure-flow cerebral bio-signals, it is difficult to compare and contrast the effectiveness of the approaches quantitatively. Moreover, the NIRS datasets in Scholkmann et al. and Hayashi et al. [59,65] were obtained from experimental recordings in healthy subjects, whereas Eames et al. [64] analyzed CBFV data from a small cohort of acute stroke patients and one healthy control, limiting generalizability.

It should also be noted that Scholkmann et al. and Eames et al. applied interpolation to correct distorted signal segments rather than to reconstruct missing data [64,65], whereas Hayashi et al. used interpolation to synthesize training data for fine-tuning classification models [59]. These studies, therefore, employed interpolation for signal correction and data augmentation, not for estimating and recovering segments of data that were completely lost. This fundamental difference in the interpolation objective highlights the critical knowledge gap in interpolation strategies for missing segments in pressure-flow cerebral bio-signals.

### 3.3. Combined Interpolation and Artifact Correction/Signal Reconstruction Approaches

Two studies were identified that employed combined approaches integrating interpolation with artifact correction and signal reconstruction techniques: wavelet-based correction and filtering [35,60]. Both studies used fNIRS datasets to validate artifact correction and reported experimental results comparing interpolation alone against combined interpolation with artifact correction/signal reconstruction approaches. Spline interpolation was employed to correct baseline shifts by both studies [35,60], while Jahani et al. also used it for spike correction [60]. Cubic spline interpolation was utilized by Gao et al. to correct severe oscillation artifacts [35]. Although both studies employed interpolation approaches, they did not apply them to recover missing segments but rather used them to correct the artifact-riddled signal segments [35,60]. However, these studies represent applications of interpolation for the correction of signal distortions, rather than for the estimation or reconstruction of completely missing data segments. Therefore, this highlights the persistent knowledge gap in interpolation strategies for missing segments in pressure-flow cerebral bio-signals.

From these studies, summarized information regarding the subjects, signal types, and measurements, missing data/artifacts characteristics, and detection techniques used, sensor location, and interpolation strategy utilized are presented in Table 2 for a comprehensive overview. Overall, the studies summarized in Table 2 demonstrate that interpolation has been applied exclusively to fNIRS-based cerebral oxygenation signals, using broadly similar sensor configurations and experimental setups, and has consistently been embedded within broader motion-artifact detection and correction pipelines rather than used as a standalone strategy to address missing pressure-flow data segments.

### 3.4. Comparative Studies with Interpolation Approaches

Three studies have conducted comparative evaluations of the spline interpolation method [61,62,63] for motion artifact correction, comparing their performance with other artifact correction and signal reconstruction techniques such as Wavelet Filtering, Principal Component Analysis (PCA), Targeted Principal Component Analysis (tPCA), and Kalman Filtering.

These three studies employed the MARA proposed by Scholkmann et al. and utilized fNIRS datasets for comparative analysis [65]. A comprehensive overview of these three studies is presented in Table 3, summarizing information regarding the subjects, signal types and measurements, characteristics of missing data and artifacts, detection techniques used, sensor location, methods compared, and the interpolation techniques employed.

All included studies in Table 3 applied the MARA spline interpolation algorithm, focusing primarily on motion-artifact detection and correction in fNIRS-based cerebral oxygenation data acquired from similar healthy populations using comparable sensor configurations. As highlighted previously, the MARA approach applied spline interpolation to correct distorted signal segments rather than to reconstruct missing signal segments, further underscoring the persistent knowledge gap in the application of interpolation strategies for missing segments in pressure-flow cerebral bio-signals.

**Figure 3:**
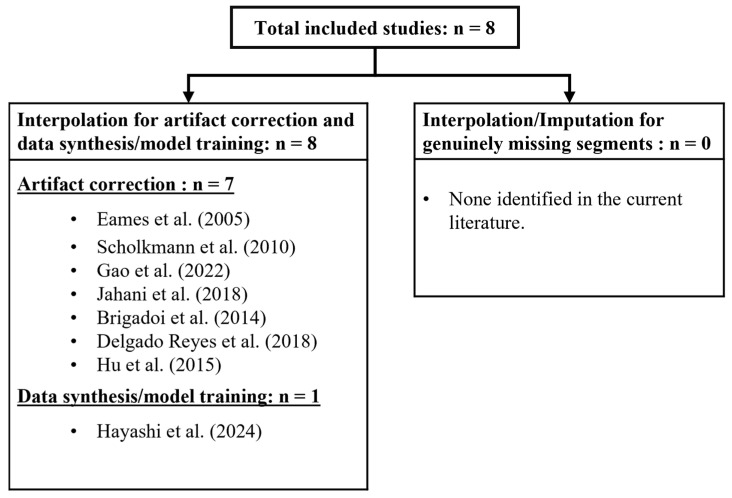
Classification of interpolation and imputation approaches in the included studies [35,59,60,61,62,63,64,65].

Across the eight studies included in this review, none applied interpolation methods to reconstruct missing segments in continuous pressure-flow cerebral bio-signals. Instead, the techniques were mainly used to correct signal distortions or to generate data for training models. This distinction is summarized in Figure 3, which classifies the included studies by their utilization of interpolation and shows that no study in the current literature utilizes interpolation and imputation strategies for the reconstruction of genuinely missing segments. These consistent findings underscore a critical knowledge gap in the current literature, as no existing study has systematically addressed applications of interpolation methods on estimating or restoring missing pressure-flow cerebral bio-signals. The following Discussion Section examines the clinical and methodological implications of this knowledge gap and proposes directions for future research for the interpolation and imputation strategies for missing segments in continuous pressure-flow cerebral bio-signals.

## 4. Discussion

Maintaining the continuity and physiological plausibility of pressure-flow cerebral bio-signals is critically important in scenarios when uninterrupted high-resolution signal streams are required for either physiologic event forecasting or for derivation of more complex cerebrovascular physiologic metrics to guide individualized therapeutic strategies in neurocritical care [38,39,40,41]. These pressure-flow cerebral bio-signals are acquired as sensor measurements and are therefore prone to common sensor malfunction pitfalls such as transient device disruptions, sensor detachment, motion artifacts, or calibration drift, which can cause missing or unusable signal segments. In such scenarios, simply discarding missing or unusable signal segments is not a viable option. As every segment of valid data is scarce and valuable, preserving continuity is critical to ensure the stable computation of cerebral autoregulatory indices. Therefore, missing and unusable signal segments must be filled with the most plausible physiological values that approximate what the pressure-flow cerebral bio-signal would have been at those time segments.

Interpolation and imputation strategies provide a critical methodological framework for reconstructing missing signal segments and restoring signal continuity while theoretically offering physiological plausibility and preserving cerebrovascular temporal dynamics. However, the findings of this systematic scoping review reveal that no study has applied or systematically evaluated imputation methods for recovering missing segments in pressure-flow cerebral bio-signals, and none of the included studies directly addressed interpolation strategies for reconstructing missing segments in pressure-flow cerebral bio-signals where no underlying pressure-flow physiological signal data remain. Instead, all included studies in this review either employed interpolation techniques such as cubic spline, linear interpolation, and possibilistic membership-based methods as standalone [59,64,65] or combined approaches [35,60] to correct distorted signal segments rather than reconstruct missing data [35,60,64,65] or to synthesize training data for fine-tuning classification models [59]. This highlights the fundamental knowledge gap that interpolation and imputation strategies for missing segments in pressure-flow cerebral bio-signals remain critically understudied.

Within this context, studies that primarily focused on using interpolation for simulated artifact correction [35,65] as well as modeling and synthesizing pressure-flow data [59] were included because their methods detect and replace non-physiological data in continuous pressure-flow cerebral bio-signals by interpolating between neighboring artifact-free data points in time. In doing so, they exploit the local temporal structure of the signal to reconstruct artifactual segments, thus providing relevant insight into interpolation strategies for handling unusable segments that are effectively treated as missing in the analysis pipeline. In addition, these studies provide insight into how interpolation methods can be evaluated, as they introduce simulated artifacts into real data and then compare the interpolated (corrected) signal segments directly against the original artifact-free segments.

From a technical and clinical perspective, ensuring the continuity and reliability of high-frequency, multi-channel pressure-flow cerebral bio-signals is critical when deriving cerebral autoregulatory metrics and guiding individualized therapy in neurocritical care. The inability to meaningfully reconstruct missing data segments risks undermining the validity of continuous indices (e.g., pressure reactivity index, oxygenation-based bio-markers), and individualized metrics such as CPPopt, MAPopt, iICP, and BISopt, particularly in vulnerable patient groups such as acute stroke, traumatic brain injury, and other neurocritical care populations, as well as neonatal and pediatric patients undergoing cerebrovascular monitoring. Thereby, this may restrict the development and translation of clinical research algorithms into real-time clinical decision-making tools that focus on optimal blood pressure targets, adjustment of sedation and ventilation, and escalation or de-escalation of perfusion-supportive or neuroprotective therapies at the bedside [16,17,18,19,20].

Although this review specifically focuses on interpolation and imputation strategies for pressure-flow cerebral bio-signals, in the broader literature, a wide range of methodologies, including traditional approaches: linear, spline, and polynomial interpolation [44,45,46], statistical imputation techniques [46], model-based estimation frameworks [49], machine learning and deep learning methods [50,51] have shown promise for reconstructing and recovering missing segments in general time series applications by modeling complex non-linear relationships and capturing long-range temporal dependencies [50,67,68,69,70,71]. Nevertheless, none of these approaches have been applied to recovering missing segments in continuous pressure-flow cerebral bio-signals, highlighting that domain-specific research has not been updated with methodological advancements in interpolation and imputation strategies.

This discrepancy may be caused by several factors: the relative scarcity of large, annotated pressure-flow cerebral bio-signal datasets, limited research and development in continuously updating real-time neurocritical care algorithms suitable for bedside implementation, limited real-time pressure-flow cerebral bio-signal utilization in experimental studies of neuroscience and psychology, and insufficient recognition of missing segments reconstruction as a distinct research problem separate from artifact correction. Therefore, this systematic scoping review provides a foundational guide for understanding the existing knowledge gaps, limitations, and avenues for the development and validation of interpolation and imputation strategies for missing segments in continuous pressure-flow cerebral bio-signal.

### 4.1. Limitations of Literature

There are several methodological limitations that exist in the current literature that could have led to a critical knowledge gap in interpolation and imputation strategies for missing segments in pressure-flow cerebral bio-signals. First, the exclusive focus on artifact correction methodologies rather than recovering the missing segments fundamentally limits the utilization of interpolation and imputation strategies for continuous pressure-flow cerebral bio-signals in scenarios where signal segments are genuinely absent rather than distorted or contaminated. Additionally, the lack of studies that quantitatively evaluate the incidence of genuinely absent data due to sensor-related issues, or signals that are irretrievably contaminated, as well as recoverable contaminated signals, further underscores this critical knowledge gap. Secondly, the majority of studies aimed at deriving neurocritical care metrics and indices have been conducted as observational studies that rely on post-processed data [18,19,20]. In these studies, missing signal segments were typically discarded [18,19,20]. This limitation is reflected in our review, where no dedicated interpolation/imputation strategies whose primary goal is reconstructing missing ICP segments were identified, despite ICP being a central signal in neurocritical care. This is because many ICP-related indices are currently computed as post-processing metrics and are designed to tolerate such gaps rather than explicitly reconstruct missing segments, and their real-time implementation at the bedside remains limited [30,72]. Furthermore, the translation of findings of these clinical studies into real-time, continuously derived cerebrovascular metrics and autoregulatory indices has not yet been achieved [19,20]. Similarly, in non-clinical applications, pressure-flow cerebral bio-signals (e.g., NIRS and fNIRS) were widely used in experimental neuroscience and psychological research to study neurovascular coupling and task-evoked hemodynamic responses. However, these signals were typically analyzed offline, with missing or corrupted signal segments removed during preprocessing rather than explicitly interpolated or imputed [31,73]. Lastly, the lack of annotated pressure-flow cerebral bio-signal datasets and simulated datasets, which fail to reflect the quantitative characteristics of real-world missing data in terms of gaps, duration, and causes, and the fact that such missingness can affect the accuracy of clinical indices, the quality of medical decision-making, and associated healthcare costs, further hinders the utilization of interpolation and imputation techniques. As a result, our characterization of the knowledge gap is qualitative rather than quantitative, which limits our ability to estimate the clinical impact of missing segments at the patient or population level in this review. Collectively, all these limitations have led to the failure to identify and address genuinely missing signal segments as a distinct research question. Therefore, the limited motivation to treat genuinely missing signal segments as a distinct research problem, combined with the above-mentioned constraints, has so far discouraged the use of data and computation-intensive deep learning architectures (e.g., RNNs, LSTMs, GANs, and Transformers) for interpolation and imputation of missing segments in cerebral pressure-flow bio-signals, as these models typically require extensive training and validation with substantial computational resources. Apart from these limitations, another limitation of the literature is that the computational efficiency of the included interpolation strategies could not be systematically evaluated, as only one study reported hardware specifications and algorithm run time. As a result, this review was unable to provide a comparative analysis of computational efficiency across interpolation approaches, despite its importance for real-time neurocritical care applications, as resource-constrained portable hardware platforms are used at bedside for real-time computation.

### 4.2. Limitations of Review

There were several methodological limitations associated with this systematic scoping review. This study only reviewed studies published before 16 June 2025; thus, this may not reflect the most recent studies published afterward. There is a potential for language bias in this review, as this review only includes studies published in the English language. The search strategy was restricted to five electronic databases (Biosis, Scopus, Embase, PubMed, and the Cochrane Library), which may have missed relevant studies published in gray literature sources. The original search strategy was developed to capture all cerebral bio-signals and was subsequently divided into separate electrophysiological and pressure-flow systematic reviews, given the heterogeneous nature of cerebral bio-signals and the sheer volume of the yielded results. However, because the initial search and first-stage screening retained all studies proposing methods to impute or interpolate missing segments in any cerebral bio-signal, applying a priori filtering based on signal type at the full-text stage to extract cerebral pressure-flow bio-signals minimized the risk of selection bias. Nevertheless, if the initial search strategy had focused solely on cerebral pressure-flow bio-signals, it would likely have yielded fewer studies to be screened. Although all articles included in this review were published in peer-reviewed academic journals, a formal, systematic risk of bias assessment was not performed, as the primary aim of this work was a methodological scoping synthesis rather than an evaluation of clinical or epidemiological outcomes. Therefore, the reported performance of the interpolation and imputation strategies, as well as the conclusions synthesized from these studies, may be influenced by biases in the datasets, methodological flaws, or limitations in the reproducibility and generalizability of the proposed strategies. In addition, we excluded studies based solely on simulated pressure-flow cerebral bio-signals. Although simulation studies are useful for algorithm testing, this choice limits our synthesis to interpolation and imputation strategies evaluated on real data and may omit algorithms/methods that have so far been assessed only in simulated settings.

### 4.3. Recommendations and Future Directions

Future research should prioritize quantitative assessments of the incidence of missing signal segments (sensor-related issues or signals that are irretrievably contaminated) versus recoverable contaminated signal segments in continuous pressure-flow cerebral bio-signals. This approach will quantitatively highlight the importance of recovering genuinely missing signal segments and lay the foundation to identify that recovering genuinely missing signal segments in continuous pressure-flow cerebral bio-signals through interpolation and imputation strategies is a distinct research question. Also, establishing clear definitions and frameworks for identifying missing and irretrievably contaminated signal segments vs. recoverable contaminated signal segments will pave the way for frameworks and guidelines for when to use interpolation and imputation strategies and artifact correction methodologies, respectively, in clinical settings, as well as non-clinical settings such as experimental neuroscience and psychology.

Future research should also focus on utilizing existing interpolation and imputation strategies for pressure-flow cerebral bio-signals and validating the physiological plausibility of reconstructed missing signal segments. Then, these interpolation and imputation strategies should be enhanced, and novel interpolation/imputation strategies should be developed, specifically focusing on the recovery of missing segments in pressure-flow cerebral bio-signals by incorporating physiologically informed cerebrovascular dynamics, autoregulation, and expected signal patterns. Second, the availability of publicly accessible, large, and annotated pressure-flow cerebral bio-signal datasets will accelerate research in cerebral multimodal monitoring, and such datasets will establish the foundational infrastructure required to advance studies on missing signal recovery and artifact correction. Third, the establishment of standardized evaluation frameworks with physiological validity assessments will facilitate clear guidelines for evaluating signal quality of interpolated or imputed missing segments in pressure-flow cerebral bio-signals. This will ensure clinical validity, safety, effectiveness, reliability, and transparency of the clinical research. Finally, future work should focus on the design of advanced interpolation and imputation algorithms optimized for time and space complexity and should be computationally efficient to be deployed in resource-constrained hardware platforms, enabling real-time deployment in clinical environments.

## 5. Conclusions

This systematic scoping review was conducted to comprehensively evaluate interpolation and imputation strategies for missing segments in continuous pressure-flow cerebral bio-signals. The results revealed that no study had systematically addressed the interpolation and imputation strategies for missing segments in pressure-flow cerebral bio-signals. All included studies in this review employed interpolation techniques to correct distorted signal segments or to synthesize data, rather than to reconstruct genuinely missing segments. The exclusive focus on artifact correction, the lack of standardized definitions for missing segments, and the lack of quantitative assessments of the incidence of missing signal segments versus recoverable contaminated signal segments result in a failure to recognize the recovery and reconstruction of genuinely missing signal segments using interpolation or imputation as a distinct research question. Taken together, this systematic scoping review provides the first structured synthesis of interpolation and imputation strategies for missing segments in continuous pressure-flow cerebral bio-signals and explicitly identifies a fundamental knowledge gap that no study has systematically addressed in reconstructing genuinely missing segments in these signals. By synthesizing the existing literature on how continuous pressure-flow cerebral bio-signals are utilized and processed, this review also identifies the underlying limitations, methodological constraints, and data gaps that contribute to this knowledge gap. Building on these findings, this review emphasizes the need for future research and provides a structured foundation for a future methodological research agenda focused on developing and validating interpolation and imputation strategies for missing segments in continuous pressure-flow cerebral bio-signals.

## Figures and Tables

**Table 1:** Summary of standalone interpolation approaches.

Reference	Subject Information	Signal Type, Sampling Rate, and Measurements	Missing Data Characteristics and the Detection Technique Used	Sensor Location	Utilization of Interpolation Techniques
Eames et al. (2005) [64]	7 acute stroke patients and one healthy control (All male, age range 52–87 years)	CBFV 0.2 Hz NIBP 200 Hz ECG 200 Hz Transcutaneous CO_2_ 200 Hz	Ectopic heartbeats are treated as naturally occurring artifacts to BP and CBFV signals. Ectopic heartbeats were detected and marked manually in the CBFV data.	CBFV: Middle cerebral arteries were insonated bilaterally. NIBP: Middle finger of the left hand is used for non-invasive blood pressure. ECG: three-lead surface ECG sensor setup used.	Linear interpolation was used to remove narrow spikes on the CBFV signals.
Hayashi et al. (2024) [59]	3 healthy subjects. Generated a dataset of 30 experimental trials.	HbO_2_, HHb NIRS 10.20 Hz Wavelength: 770–840 nm	This proposed approach does not utilize a method to detect and localize artifacts in NIRS data. This proposed approach is triggered when the given NIRS signals are misclassified.	Prefrontal areas	The proposed pdi-Bagging algorithm adds virtual sample data generated by possibilistic membership-based interpolation around misclassified instances in model training.
Scholkmann et al. (2010) [65]	3 NIRS datasets with simulated motion artifacts were used. 1st dataset: short impulses 2nd dataset: base shifts 3rd dataset: temporally limited low-frequency oscillations	HbO_2_, HHb Laboratory-developed Multi-distance Near-Infrared Spectroscopy instrument [66] Sampling rate: 10 Hz	Motion artifact (MA) segments are detected by moving the standard deviation with a user-specified threshold and marked.	Motor cortex (3rd dataset)	Spline interpolation was utilized to correct signal segments with motion artifacts.

BP = Blood Pressure, CBFV = Cerebral Blood Flow Velocity, ECG = Electrocardiogram, HbO_2_ = Oxyhemoglobin, HHb = Deoxyhemoglobin, NIBP = Non-Invasive Blood Pressure, NIRS = Near-Infrared Spectroscopy, pdi-Bagging = Possibilistic Data Interpolation-Bagging.

**Table 2:** Summary of combined interpolation and artifact correction/signal reconstruction approaches.

Reference	Subject Information	Signal Type, Sampling Rate, and Measurements	Missing Data Characteristics and the Detection Technique Used	Sensor Location	Utilization of Interpolation Techniques
Gao et al. (2022) [35]	40 healthy participants (16 male and 24 females, mean age 32 years) Simulated artifacts: baseline shifts and oscillatory (severe and slight) artifacts added to fNIRS data.	HbO_2_, HHb, TSI 10 Hz fNIRS	An fNIRS-based detection strategy is used to detect motion artifacts utilizing two-sided moving standard deviation and dynamic threshold. By using these techniques, baseline shifts and oscillatory (severe and slight) artifacts were detected.	Symmetrically on the left and right forehead	Interpolation techniques and filtering were used to correct detected artifact types. Severe oscillations: cubic spline interpolation Baseline shifts: spline interpolation Slight oscillations: Dual-threshold wavelet-based filtering Finally, the data was passed through a high-pass filter.
Jahani et al. (2018) [60]	Dataset I: 7 healthy adults Subjects performed specific movements of reading aloud, nodding head up/down or sideways, twisting upper body, rapidly shaking head, raising eyebrows. Dataset II: 5 healthy subjects in the resting state. (Assumed this dataset contained a minimum number of motion artifacts).	Optical Density Signals, HbO_2_, HHb 50 Hz fNIRS	Motion artifacts are detected for outlier detection in baseline shifts and spikes by evaluating the SNR of the signal with a pre-configured threshold. Then, baseline shifts are corrected by applying spline interpolation, and spike removal is done with a de-noising algorithm.	Frontal lobe	Spline interpolation was utilized to correct baseline shifts, while Savitzky–Golay filtering served as a de-noising algorithm.

fNIRS = Functional Near-Infrared Spectroscopy, HbO_2_ = Oxyhemoglobin, HHb = Deoxyhemoglobin, SNR = Signal-to-Noise Ratio, TSI = Tissue Saturation Index.

**Table 3:** Summary of comparative studies with interpolation approaches.

Reference	Subject Information	Signal Type, Sampling Rate, and Measurements	Missing Data Characteristics and the Detection Technique Used	Sensor Location	Methods Compared	Utilization of Interpolation Methods
Brigadoi et al. (2014) [61]	22 healthy subjects (10 males, mean age 25.54 ± 3.14 years) 4 recordings were discarded, thus 18 participants were used for the final analysis.	Optical Density of HbO_2_ and HHb Sampling frequency is approximately 7.8 Hz fNIRS Wavelength : 690–830 nm	Motion artifacts were detected using the hmrMotionArtifact algorithm from Homer2 with pre-configured parameters. For the spline method, hmrMotionArtifactByChannel was used to detect motion artifacts. The experiment was designed to generate artifacts mainly from jaw movement induced by the vocal response during the experiment.	Frontal and Pre-motor regions	Spline Interpolation PCA Wavelet Filtering CBSI Kalman Filter Trial rejection raw non-corrected data	Spline interpolation was utilized to correct signal segments affected by motion artifacts, as employed by Scholkmann et al. [65].
Delgado Reyes et al. (2018) [62]	Total of 25 healthy children in the population 11 with a mean age of 3.5 years (SD = 0.06) 14 with a mean age of 4.5 years (SD = 0.08)	Optical Density, HbO_2_, HHb, HbT 50 Hz fNIRS Wavelength: 690–830 nm	Artifact detection was done using the hmrMotionArtifact and hmrMotionArtifactByChannel algorithms in Homer2. Used two sets of parameters (Original and Relaxed parameters) for Homer2 functions.	Bilateral frontal, temporal, and parietal cortices	PCA tPCA Spline Wavelet CBSI	Spline interpolation was utilized to correct signal segments affected by motion artifacts, as employed by Scholkmann et al. [65].
Hu et al. (2015) [63]	12 healthy children 8 females age range 6.8–12.6 Mean age ± SD = 9.9 ± 1.75 years	Optical Density 10 Hz fNIRS Wavelength: 690 nm and 830 nm	Motion artifacts were categorized into four types: Type A: spikes with a standard deviation of 50 over the mean within one second (sudden move generated motion artifact) Type B: Peaks with a standard deviation of 100 from the mean during a time portion ranging from 1 to 5 s Type C: Gentle slope between 5 and 30 s with a standard deviation of 300 from the mean Type D: Slow baseline shifting longer than 30 s with a standard deviation of 500 from the mean	Left inferior frontal gyri (language-related area)	Spline Wavelet PCA Moving average (MA) CBSI Wavelet + MA	Spline interpolation was utilized to correct signal segments affected by motion artifacts, as employed by Scholkmann et al. [65].

CBSI = Correlation-Based Signal Improvement, fNIRS = Functional Near-Infrared Spectroscopy, HbO_2_ = Oxyhemoglobin, HbT = Total Hemoglobin, HHb = Deoxyhemoglobin, MA = Moving Average, PCA = Principal Component Analysis, SD = Standard Deviation, tPCA = Targeted Principal Component Analysis.

## Data Availability

All relevant data are contained in the manuscript and the extraction tables provided in the Supplementary Materials (Tables S2–S4) or are publicly available at their respective data source.

## Supplementary Materials

The following supporting information can be downloaded at: https://www.mdpi.com/article/10.3390/s26103134/s1. Supplementary Materials are provided in the Supplementary Files S1, S2 and S3.
