# Supplementary material for: Interpolation and Imputation Strategies for Missing Segments in Continuous Pressure-Flow Cerebral Bio-Signals: A Systematic Scoping Review
Source: Sensors (Basel). 2026 May 15;26(10):3134. doi: 10.3390/s26103134 (PMC13210763; doi:10.3390/s26103134)
Supplement: Supplementary file 1 [file sensors-26-03134-s001.zip › sensors-4289789-supplementary.pdf]

Supplementary File S1

Table S1. PRISMA ScR Checklist

Section and Topic	Item #	Checklist item	Location where item is reported
**Title**			
Title	1	Identify the report as a systematic review.	Pg. 1 (Title)
**Abstract**			
Abstract	2	See the PRISMA 2020 for Abstracts checklist.	Pg. 1-2 (Abstract)
**Introduction**			
Rationale	3	Describe the rationale for the review in the context of existing knowledge.	Pg. 2 - 3 (Section 1)
Objectives	4	Provide an explicit statement of the objective(s) or question(s) the review addresses.	Pg. 3 (Section 1)
**Methods**			
Eligibility criteria	5	Specify the inclusion and exclusion criteria for the review and how studies were grouped for the syntheses.	Pg. 5 - 6 (Section 2.2)
Information sources	6	Specify all databases, registers, websites, organisations, reference lists and other sources searched or consulted to identify studies. Specify the date when each source was last searched or consulted.	Pg. 6 (Section 2.3)
Search strategy	7	Present the full search strategies for all databases, registers and websites, including any filters and limits used.	Pg. 6 (Section 2.3) + Supplementary File S2
Selection process	8	Specify the methods used to decide whether a study met the inclusion criteria of the review, including how many reviewers screened each record and each report retrieved, whether they worked independently, and if applicable, details of automation tools used in the process.	Pg. 6 - 7 (Section 2.4)
Data collection process	9	Specify the methods used to collect data from reports, including how many reviewers collected data from each report, whether they worked independently, any processes for obtaining or confirming data from study investigators, and if applicable, details of automation tools used in the process.	Pg. 7 (Section 2.5)
Data items	10a	List and define all outcomes for which data were sought. Specify whether all results that were compatible with each outcome domain in each study were sought (e.g. for all measures, time points, analyses), and if not, the methods used to decide which results to collect.	Pg. 7 (Section 2.5)

	10b	List and define all other variables for which data were sought (e.g. participant and intervention characteristics, funding sources). Describe any assumptions made about any missing or unclear information.	N/A
Study risk of bias assessment	11	Specify the methods used to assess risk of bias in the included studies, including details of the tool(s) used, how many reviewers assessed each study and whether they worked independently, and if applicable, details of automation tools used in the process.	All articles published in academic journals, as such, biases were assumed to have been screened
Effect measures	12	Specify for each outcome the effect measure(s) (e.g. risk ratio, mean difference) used in the synthesis or presentation of results.	N/A
Synthesis methods	13a	Describe the processes used to decide which studies were eligible for each synthesis (e.g. tabulating the study intervention characteristics and comparing against the planned groups for each synthesis (item #5)).	N/A
	13b	Describe any methods required to prepare the data for presentation or synthesis, such as handling of missing summary statistics, or data conversions.	N/A
	13c	Describe any methods used to tabulate or visually display results of individual studies and syntheses.	All data items for each method were summarized and tabulated and are included in Supplementary Tables S2-S4.
	13d	Describe any methods used to synthesize results and provide a rationale for the choice(s). If meta-analysis was performed, describe the model(s), method(s) to identify the presence and extent of statistical heterogeneity, and software package(s) used.	N/A
	13e	Describe any methods used to explore possible causes of heterogeneity among study results (e.g. subgroup analysis, meta-regression).	N/A
	13f	Describe any sensitivity analyses conducted to assess robustness of the synthesized results.	N/A
Reporting bias assessment	14	Describe any methods used to assess risk of bias due to missing results in a synthesis (arising from reporting biases).	Pg. 16 – 17 (Section 4.1)

Certainty assessment	15	Describe any methods used to assess certainty (or confidence) in the body of evidence for an outcome.	N/A
**Results**			
Study selection	16a	Describe the results of the search and selection process, from the number of records identified in the search to the number of studies included in the review, ideally using a flow diagram.	Pg. 8 – 9 (Section 3.1)
	16b	Cite studies that might appear to meet the inclusion criteria, but which were excluded, and explain why they were excluded.	N/A
Study characteristics	17	Cite each included study and present its characteristics.	Pg. 8 - 14 (Section 3.1 - 3.4)
Risk of bias in studies	18	Present assessments of risk of bias for each included study.	All articles included in this review were published in academic journals, and thus their biases were assumed to have been screened as part of the independent peer-review processes of the respective journals. However, no formal risk-of-bias assessment was conducted as part of this scoping review.
Results of individual studies	19	For all outcomes, present, for each study: (a) summary statistics for each group (where appropriate) and (b) an effect estimate and its precision (e.g. confidence/credible interval), ideally using structured tables or plots.	Pg. 10 - 11 Tables 1, Pg. 12 Table 2 and Pg. 13 Table 3. + Supplementary File S3 Supplementary Tables S2, S3 and S4.
Results of syntheses	20a	For each synthesis, briefly summarise the characteristics and risk of bias among contributing studies.	All articles included in this review were published in academic journals, and thus their biases were assumed to

			have been screened as part of the independent peer-review processes of the respective journals. However, no formal risk-of-bias assessment was conducted as part of this scoping review.
	20b	Present results of all statistical syntheses conducted. If meta-analysis was done, present for each the summary estimate and its precision (e.g. confidence/credible interval) and measures of statistical heterogeneity. If comparing groups, describe the direction of the effect.	No statistical synthesis conducted
	20c	Present results of all investigations of possible causes of heterogeneity among study results.	Pg. 8 - 14 (Section 3.1 - 3.4)
	20d	Present results of all sensitivity analyses conducted to assess the robustness of the synthesized results.	Pg. 8 - 14 (Section 3.1 - 3.4)
Reporting biases	21	Present assessments of risk of bias due to missing results (arising from reporting biases) for each synthesis assessed.	Pg. 17 – 18 (Section 4.2)
Certainty of evidence	22	Present assessments of certainty (or confidence) in the body of evidence for each outcome assessed.	N/A
**DISCUSSION**			
Discussion	23a	Provide a general interpretation of the results in the context of other evidence.	Pg. 14 - 16 (Section 4)
	23b	Discuss any limitations of the evidence included in the review.	Pg. 16 - 17 (Section 4.1)
	23c	Discuss any limitations of the review processes used.	Pg. 17 (Section 4.2)
	23d	Discuss implications of the results for practice, policy, and future research.	Pg. 17 - 18 (Section 4.3)
**OTHER INFORMATION**			
Registration and protocol	24a	Provide registration information for the review, including register name and registration number, or state that the review was not registered.	Review was not registered
	24b	Indicate where the review protocol can be accessed, or state that a protocol was not prepared.	Protocol not prepared

	24c	Describe and explain any amendments to information provided at registration or in the protocol.	N/A
Support	25	Describe sources of financial or non-financial support for the review, and the role of the funders or sponsors in the review.	Pg. 19 (Funding)
Competing interests	26	Declare any competing interests of review authors.	Pg. 19 (Conflicts of Interest)
Availability of data, code and other materials	27	Report which of the following are publicly available and where they can be found: template data collection forms; data extracted from included studies; data used for all analyses; analytic code; any other materials used in the review.	N/A

Supplementary File S2

The following search string was used to search five electronic databases (BIOSIS, SCOPUS, EMBASE, PubMed, and Cochrane Library) examining keywords, abstracts, and titles indexed in these databases. This search was designed to capture all methods developed for filling missing signal segments in all types of cerebral bio-signals. In the search string, pressure-flow cerebral bio-signal terms are highlighted in bold text to clearly indicate the terms specific to this systematic scoping review on interpolation and imputation strategies for missing segments in pressure-flow cerebral bio-signals.

("AMP" OR **"ARI"** OR **"arterio-jugular oxygen content "** OR **"Arterio–venous difference"** OR **"arteriovenous difference in oxygen**" OR **"Autonomics"** OR **"Autoregulation index"** OR **"Autoregulatory reserve"** OR **"AVDO2"** OR "BIS" OR "Bispectral " OR **"Blood Flow Velocities"OR "Blood Flow Velocity"** OR **"Bowman perfusion"** OR **"Brain Autoregulation "** OR "**Brain Blood Flow** " OR "**Brain metabolism**" OR "**Brain metabolites**" OR "**Brain Perfusion**" OR "**brain tissue oxygen tension**" OR "**Brain tissue oxygenation**" OR "**CA**" OR "**CBF**" OR "**CBFV**" OR "**CBFx**" OR "**CBV**" OR "**Cerebral artery flow velocity**" OR "**Cerebral Autoregulation**" OR "**Cerebral Blood Flow**" OR "**Cerebral Blood Flow Index** " OR "**Cerebral blood flow velocity**" OR "**Cerebral blood volume**" OR "**Cerebral circulation**" OR "**Cerebral Flow metabolism**" OR "**Cerebral hemodynamics**" OR "**Cerebral homeostasis**" OR "**Cerebral metabolic rate for oxygen** " OR "**Cerebral metabolism**" OR "**Cerebral microcirculation**" OR "**Cerebral microdialysis**" OR "**Cerebral Oximetry index** " OR "**Cerebral Oximetry Index** " OR "**Cerebral perfusion**" OR "**Cerebral Perfusion Pressure**" OR "**Cerebral pressure**" OR "**Cerebral pressure autoregulation**" OR "**Cerebral spinal reserve capacity**" OR "**Cerebral vascular reactivity**" OR "**Cerebral vascular resistance**" OR "**Cerebral vasculature**" OR "**Cerebral vasoconstriction**" OR "**Cerebral vasodilation**" OR "**Cerebral vasomotor responsiveness**" OR "**Cerebral Vasoreactivity**" OR "**Cerebral Vessel Diameter**" OR "**Cerebrovascular autoregulation**" OR "**Cerebrovascular control**" OR "**Cerebrovascular Function**" OR "**Cerebrovascular reactivity**" OR "**CMD**" OR "**CMRO2**" OR "**Compensatory reserve** " OR "**Cortical blood flow**" OR "**Cortical laser doppler**" OR "**Cortical perfusion** " OR "**COx**" OR "**COx-a**" OR "**CPP**" OR "**Dax**" OR "**DCS**" OR "dEEG" OR "dense array electroencephalography" OR "**Deoxyhemoglobin Index** " OR "**Diastolic flow index**" OR

"**Diastolic Flow Index** " OR "**Diffuse Correlation Spectroscopy**" OR "**Diffusion Weighted Imaging index**" OR "**DWI**" OR "**Dx**" OR "**Dx-a**" OR "**Dynamic autoregulatory index**" OR "EcOG" OR "EEG" OR "Electrocortical activity" OR "Electrocorticography" OR "Electroencephalography" OR "Electrophysiological monitoring" OR "Electrophysiology" OR "**End-tidal CO2** " OR "**EtCO2**" OR "**Flow velocity** " OR "**FV**" OR "**HbOx** " OR "**HbOx-a**" OR "**HBx**" OR "**Hbx-a**" OR "**Heart rate**" OR "**Hemedex**" OR "**hemoglobin volume index**" OR "**Hemoglobin Volume Index** " OR "**HVx**" OR "**ICP**" OR "**Induced Pressure Reactivity Index** " OR "**Intracranial pressure**" OR "**iPRx**" OR "**Jugular bulb saturation** " OR "**Jugular venous oxygen saturation**" OR "**Kety–Schmidt technique**" OR "**L-PRx**" OR "**Laser Doppler flowmetry** " OR "**Laser-Doppler Index** " OR "**LAx**" OR "**LDF**" OR "**LDx**" OR "**Licox**" OR "**Long Pressure Reactivity Index** " OR "**Low-Frequency Autoregulation Index** " OR "**Lx**" OR "**Lx-a**" OR "**Mean flow index**" OR "**Mean Flow Index** " OR "**Mean transit time** " OR "**Median arterial pressure**" OR "**MTT**" OR "**Multi-scale entropy**" OR "**Mx**" OR "**Mx-a**" OR "**Near infrared spectroscopy** " OR "**Near-infrared spectrometry**" OR "**Neurovascular autoregulation**" OR "**Neurovascular coupling**" OR "**Neurovascular reactivity**" OR "**Neurovent-PTO**" OR "**NIRS**" OR "**OEF**" OR "**OHT**" OR "**orthostatic hypotension test** " OR "**ORx**" OR "**oxygen extraction fraction** " OR "**Oxygen Reactivity Index** " OR "**Oxyhemoglobin Index** " OR "**Parenchymal brain tissue oxygen**" OR "**Parenchymal thermal diffusion**" OR "**PAx**" OR "**PAx**" OR "**PbtO2**" OR "**Perfusion-weighted imaging index**" OR "**Peripheral oxygen saturation** " OR "**PPR**" OR "**Pressure Reactivity index**" OR "**Projection pursuit regression**" OR "**PRx**" OR "**PRx55-15**" OR "**Pulsatile Reactivity index** " OR "**Pulsatility Index**" OR "**Pulse Amplitude Index** " OR "**Pulse amplitude of ICP**" OR "**RAC**" OR "**RAP**" OR "**regional cerebral oxygen saturation**" OR "**Respiratory dynamics**" OR "**rSO2**" OR "sEEG" OR "Signal entropy" OR "**SjO2**" OR "**Spatially resolved NIRS**" OR "**SpO2**" OR "stereo electroencephalography" OR "**SvjO2**" OR "**Sx**" OR "**Sx-a**" OR "**Systolic flow index**" OR "**Systolic Flow Index** " OR "**TCCS**" OR "**TCD**" OR "**TCDT**" OR "**TCDx**" OR "**TDx**" OR "**TF**" OR "**TFA**" OR "**tHbx**" OR "**Thermal Diffusion** " OR "**Thermal diffusion catheter**" OR "**Thermal diffusion probe**" OR "**Thigh cuff deflation technique** " OR "**THRT**" OR "**THx**" OR "**THx-a**" OR "**Time to peak**" OR "**tissue hemoglobin index**" OR "**Tissue Oxygen Index** " OR "**Tissue Oxygenation index**" OR "**Total Hemoglobin Index** " OR "**Tox**" OR "**TOx** " OR "**TOx-a**" OR "**Transcranial color-coded duplex sonography** " OR "**Transcranial Doppler**" OR

"**Transcranial doppler index** " OR "**Transcranial Doppler Sonography**" OR "**Transfer function analysis**" OR "**transient hyperemic response test**" OR "**TTP**" OR "**Vascular reactivity**" OR "**Wavelet Cerebral Oximetry Index** " OR "**Wavelet Hemoglobin Volume Index** " OR "**Wavelet Pressure Reactivity Index** " OR "**wCOx**" OR "**wHVx**" OR **"wPRx**")

AND

("Data reconstruction methods" OR "Value prediction techniques" OR "Curve fitting strategies" OR "Function approximation methods" OR "Smoothing approaches" OR "Data filling techniques" OR "Signal restoration methods" OR "Missing data handling techniques" OR "Value approximation")

Supplementary File S3

Supplementary Table S2: Detailed summary of standalone interpolation approaches.

Reference	Subject Information	Signal Type, Sampling rate, and Measurements	Missing Data Characteristics and the detection technique used	Sensor Location	Interpolation Technique used	Methods Compared	Evaluating the Effectiveness of Interpolation Methods	Study Results and Conclusion	Limitations
Eames et al. (2005) [64]	7 acute stroke patients and one healthy control (all male, age range 52-87 years)	CBFV via TCD 0.2 Hz<br><br>NIBP 200 Hz<br><br>ECG 200 Hz<br><br>Transcutaneous CO_2 200 Hz	Ectopic heartbeats are treated as naturally occurring artifacts to BP and CBFV signals.<br><br>Ectopic heartbeats were detected and marked manually in the data.	CBFV: Middle cerebral arteries were insonated bilaterally.<br><br>NIBP: Middle finger of the left hand is used for non-invasive blood pressure.<br><br>ECG: three-lead surface	Linear interpolation was used to remove narrow spikes on the CBFV signals.<br><br>Computational hardware specifications were not reported.	Compared the analysis of the datasets where the raw CBFV signal with ectopic heartbeats was retained and removed.<br><br>Analysis 1: ectopic heartbeats retained.<br><br>Analysis 2: Ectopic heartbeats removed and replaced using linear interpolation.<br><br>Analysis 3: manually marked 30-second ectopic heartbeat signal dataset.<br><br>Analysis 4: Coherent averaging of raw data (raw BP and CBFV) .	Coherence between CBFV and NIBP was used as a metric.<br><br>Removal of ectopic heartbeats by linear interpolation significantly lowers coherence and gain compared to retaining the ectopic heartbeats in CBFV signals.<br><br>Impulse and step responses were similar in shape in the data where ectopic beats were retained or removed, but the peak amplitude was approximately half following ectopic removal.	Significantly lower coherence and gain after removal of ectopic heartbeats by linear interpolation in CBFV signals.<br><br>Significantly lower coherence was caused by the removal of spikes and the introduction of noise in the interpolated segments in the removed ectopic heartbeat segments.	Utilization of CBFV as a surrogate for CBF is acceptable when patients are at rest, as MCA variation is negligible.<br><br>Small sample size.<br><br>Limited validation for accuracy in the higher rates of ectopic heartbeats.<br><br>Non-invasive BP measurement is less reliable compared to invasive ABP.

				ECG sensor setup used				The study pointed to the possibility of loss of useful information in the removal and interpolation of the ectopic heartbeat segments, as ectopic heartbeats inherently contain physiological information about dynamic CA.<br><br>Assessment of dynamic cerebral autoregulation can be reliably performed in patients with up to 8% ectopic heartbeats without removing them from the data.	Linear interpolation may introduce noise and remove useful information from the CBFV signal.
Hayashi et al. (2024[59]	3 healthy subjects.<br><br>Generated a dataset of 30 experimental trials.	HbO_2, HHb<br><br>NIRS<br>10.20 Hz<br>Wavelength: 770 nm – 840 nm	This proposed approach does not utilize a method to detect and localize	Prefrontal areas	The proposed pdi-Bagging algorithm adds virtual samples data generated by possibilistic membership-based interpolation around misclassified instances in model training.	REPTree<br>AdaBoost<br>MultiBoost	The average final recognition rate for pdi-Bagging among the three participants was 93.33%.<br>This was higher than AdaBoost (92.99%), MultiBoost (92.30%), and REPTree (92.31%).	The experimental results demonstrated the effectiveness of the proposed pdi-Bagging in classifying brain activity (active/steady	Small Sample size.<br><br>Need to evaluate with real-world NIRS data and BCI application what accounts for complexities in real-world scenarios.

			artifacts in NIRS data.<br><br>This proposed approach is triggered when the given NIRS signals are misclassified.		Computational hardware specifications were not reported.		The PDI-Bagging algorithm was found to be statistically superior to REPTree and MultiBoost, and highly robust against noisy data patterns.	state) using NIRS data.<br><br>This proposed method is best suited for the classification of brain activity, but not as a pre-processing step in artifact detection and correction within the biomedical signal processing pipeline.	
Scholkmann et al. (2010) [65]	3 NIRS datasets with simulated motion artifacts were used.<br><br>1st dataset: short impulses	HbO_2, HHb<br><br>Laboratory-developed Mult-distance Near-Infrared Spectroscopy instrument [66]<br><br>Sampling rate: 10 Hz	Motion artifact (MA) segments are detected by moving the standard deviation with a user-specified threshold and marked.	Motor cortex (3rd dataset)	The proposed "Movement Artifact Reduction Algorithm (MARA)" first detects motion artifact segments in the signal by moving the standard deviation with a user-specified threshold and marks them. Then, marked motion artifact segments are corrected with Spline interpolation.	Compared against real and simulated artifact datasets with and without the MARA algorithm.	Root Mean Square Error (RMSE) and Percent Root Difference (PRD), Pearson correlation coefficient (R), and SNR are used as evaluation metrics.<br><br>Validation is performed using simulated and real NIRS datasets, where the proposed MARA algorithm yields a decrease in PRD and RMSE, and substantially increases the	The MARA approach enables semi-automatic detection and reduction of movement artifacts in NIRI signals without substantially changing the underlying physiological signals.	Dependence on manual selection of optimal parameters(W - window length, T - threshold), and p - spline parameter)<br><br>Efficacy of MARA depends on artifact variance exceeding physiological signal variance. If artifacts are

	2nd dataset: base shifts<br><br>3rd dataset: temporally limited low frequency oscillations				Computational hardware specifications were not reported.		correlation in the simulated dataset. Also, the MARA algorithm improved SNR in real NIRS dataset for evoked hemodynamic responses.	MARA has linear time complexity (O(n)) and can be implemented as a real-time Motion artifact detection and correction algorithm.	too subtle, they may not be detected and corrected.<br><br>The reconstructed time-series is always an estimation of the time-series without MAs.<br><br>The use of spline interpolation tends to cause an overshoot in the interpolated data.

ABP = Arterial Blood Pressure, BP = Blood Pressure, CBFV = Cerebral Blood Flow Velocity, CA = Cerebral Autoregulation, ECG = Electrocardiogram, HbO2 = Oxyhemoglobin, HHb = Deoxyhemoglobin, MCA = Middle Cerebral Artery, NIBP = Non-Invasive Blood Pressure, NIRS = Near-Infrared Spectroscopy, pdi-Bagging = Possibilistic Data Interpolation-Bagging, PRD = Percent Root Difference, R = Pearson correlation coefficient, RMSE = Root Mean Square Error, SNR = Signal-to-Noise Ratio, TCD = transcranial Doppler

Supplementary Table S3: Detailed summary of combined interpolation and artifact correction/signal reconstruction approaches.

Reference	Subject Information	Signal Type, Sampling rate, and Measurements	Missing Data Characteristics and the detection technique used	Sensor Location	Interpolation Technique used	Methods Compared	Evaluating the Effectiveness of Interpolation Methods	Study Results and Conclusion	Limitations
Gao et al. (2022) [35]	40 healthy participants (16 male and 24 females, mean age 32 years)<br><br>Simulated artifacts: baseline shifts and oscillatory (severe and slight) artifacts added to fNIRS data.	HbO_2, HHb, TSI<br><br>10 Hz<br><br>fNIRS	The fNIRS-based detection strategy is used to detect motion artifacts utilizing two-sided moving standard deviation and dynamic threshold.<br><br>By using these techniques, baseline shifts and oscillatory artifacts were detected	Symmetrically on the left and right forehead	The proposed algorithm is based on a hybrid approach to detect artifact types and correct them using a layered correction pipeline.<br><br>First, artifacts are detected using a two-sided moving standard deviation and a dynamic threshold and categorized as oscillations (severe and slight) and baseline shifts.<br><br>Then, the severe oscillation artifacts are corrected using cubic spline interpolation, and baseline shifts are removed using spline interpolation.<br>Afterward, slight oscillations are corrected with dual-	Wavelet-based (WB) method<br><br>Accelerometer-based artifact removal (ABAMAR)<br><br>Spline interpolation<br><br>Median filtering<br><br>Spline-Savitzky–Golay<br><br>Spline-Rloess<br><br>Combination of cubic spline interpolation with wavelet filtering	SNR, Pearson's correlation coefficient (*r*), were used as evaluation metrics by comparing the original fNIRS signals vs simulated artifact-induced fNIRS data.	The proposed hybrid method performed best in both SNR and R when compared against the seven existing algorithms.<br><br>The effectiveness of dual-threshold wavelet-based filtering is based on the Mother wavelet used.<br><br>Acceleration-based detection was less effective due to mismatches with the fNIRS signal changes, and it may	The method was tested on healthy adults during sleep; thus, the performance on other complex fNIRS datasets, such as clinical data, was unclear.<br><br>The proposed approach may not fully remove noises and artifacts from instrumental and physiological sources.

					threshold wavelet-based filtering.<br><br>Finally, data were passed through a high-pass filter.<br><br>Computational hardware specifications were not reported.			lose useful information in the data.<br><br>The proposed algorithm exhibits linear time complexity and is feasible for real-time implementation using a fixed time window approach.	
Jahani et al. (2018) [60]	Dataset I: 7 healthy adults<br><br>Subjects performed specific movements of reading aloud, nodding head up/down or sideways, twisting upper body, rapidly shaking head,	Optical Density Signals, HbO_2, HHb<br><br>50 Hz<br><br>fNIRS	Motion artifacts are detected for outlier detection in Baseline shifts and spikes by evaluating the SNR of the signal with a pre-configured threshold.<br>Then, Baseline shifts are corrected by applying Spline	Frontal lobe	Combined approach with Spline interpolation + Savitzky-Golay filtering<br><br>Computational hardware specifications were reported : 3.4-GHz CPU running Windows 7; no mention of RAM specification.	Spline Interpolation<br><br>Savitzky–Golay<br><br>Robust Locally Weighted Regression and Smoothing (Rloess)<br><br>Wavelet Filtering<br><br>tPCA<br><br>CBSI	Effectiveness in motion artifacts correction as evaluated by comparing the recovered synthetic HRF to the true HRF using metrics:<br><br>Mean-Squared Error (MSE), Peak-to-Peak Error (Ep), Square of Pearson's correlation, coefficient (R^2), AUC, Total Processing Time.<br><br>Dataset I :	The Spline+Savitzky-Golay hybrid approach was deemed the optimum method because it provided reasonable and considerable improvements in all evaluation metrics while maintaining a	The proposed approach depends on a pre-configured SNR threshold in motion artifact detection.<br><br>Small sample size and this fNIRS dataset does not have the complexities of a real-world (e.g., Clinical fNIRS) fNIRS dataset.

	raising eyebrows.<br><br>Dataset II :<br>5 healthy subjects in the resting state.<br><br>(Assumed this dataset contained a minimum number of motion artifacts).		interpolation, and spike removal is done with a de-noising algorithm.			tPCA-SG<br><br>Spline-Rloess raw signals (no correction)	Spline-Rloess provided statistically superior improvements in MSE, Ep, and R^2 compared to all other algorithms.<br>Spline-SG was the second-best-performing method; however, Spline-SG (processing time = 0.08 s) is highly computationally effective compared to Spline-Rloess (processing time = 767 s).<br><br>Dataset II :<br><br>Savitzky-Golay and Spline+Savitzky-Golay methods statistically outperformed the rest in terms of MSE and Ep (p-value < 0.01).	relatively short processing time.<br><br>The proposed Spline+Savitzky-Golay hybrid approach has linear time complexity, and it can be utilized as a real-time fNIRS motion artifacts correction method.	

ABAMAR = Accelerometer-Based Artifact Removal, AUC = Area Under the Curve, CBSI = Correlation-Based Signal Improvement, Ep = Peak-to-Peak Error, fNIRS = Functional Near-Infrared Spectroscopy, HbO2 = Oxyhemoglobin, HHb = Deoxyhemoglobin, HRF = Hemodynamic Response Function, MSE = Mean Squared Error, R = Correlation Coefficient

(Pearson's r), Rloess = Robust Locally Weighted Regression and Smoothing, SG = Savitzky–Golay, SNR = Signal-to-Noise Ratio, tPCA = Targeted Principal Component Analysis, TSI = Tissue Saturation Index

Supplementary Table S4: Detailed summary of comparative studies with interpolation approaches

**Reference**	**Subject Information**	**Signal Type, Sampling rate, and Measurements**	**Missing Data Characteristics and the detection technique used**	**Sensor Location**	**Methods Compared**	**Evaluating the Effectiveness of Interpolation Methods**	**Study Results and Conclusion**	**Limitations**
Brigadoi et al. (2014)[61]	22 healthy subjects (10 males, mean age 25.54 ± 3.14 years)<br><br>4 recordings were discarded; thus 18 participants were used for the final analysis.	Optical Density of HbO_2 and HHb<br><br>Sampling frequency is approximately 7.8 Hz<br><br>fNIRS<br><br>Wavelength: 690 nm - 830 nm	Motion artifacts were detected using the *hmrMotionArtifact* algorithm from Homer2 with pre-configured parameters.<br>For the spline method *hmrMotionArtifactByChannel* was used to detect motion artifacts.<br><br>The experiment was designed to generate artifacts mainly from jaw	Frontal and Pre-motor regions	Spline Interpolation<br>PCA (thresholds: 80% and 97% variance removal)<br><br>Wavelet Filtering (Daubechies 5)<br><br>CBSI<br><br>Kalman Filter<br><br>Trial rejection raw non-corrected data	Effectiveness was evaluated using five metrics designed to assess the physiological plausibility of the recovered Hemodynamic Response Functions.<br><br>1. Area under the curve computed on the mean HRF for the first two seconds after stimulus onset (assumed that a lower index yields better artifact correction)<br>2. Ratio between the area under the curve (AUC ratio) of the mean hemodynamic response between 2 sec and 4 sec.<br>3. Within-subject standard deviation<br>4. Between-subject standard deviation	Wavelet filtering consistently demonstrated superior performance and may become the standard for fNIRS motion artifact correction, as it reduced the area under the curve where artifacts were present in 93% of cases when no correction method was used.<br><br>This study concluded that it is	Need for motion correction techniques to be tested on many different datasets with different types of motion artifacts that reflect real-world complexities of fNIRS data before a universal approach can be accepted.<br><br>Optimal parameters for motion correction techniques affect the generalizability of the methods.<br><br>Need to utilize global performance statistics, such as mean and standard

			movement induced by the vocal response during the experiment.			5. Number of trials averaged.	always better to correct for motion artifacts than reject trials.	deviation ranking, for the evaluation metrics used.<br><br>Run-time performance of the methods is not considered.
Delgado Reyes et al. (2018) [62]	Total of 25 healthy children population<br><br>11 with a mean age of 3.5 years (SD = 0.06)<br><br>14 with a mean age of 4.5 years (SD = 0.08)	Optical Density, HbO_2, HHb, HbT<br><br>50 Hz<br><br>fNIRS<br><br>Wavelength: 690 nm - 830 nm	Artifact detection was done using the *hmrMotionArtifact* and *hmrMotionArtifactByChannel* algorithms in HomER2.<br><br>Used two sets of parameters (OriginaL and Relaxed parameters) for HomER2 functions.	bilateral frontal, temporal, and parietal cortices	• PCA<br>• tPCA<br>• Spline<br>• Wavelet<br>• CBSI	The effectiveness of the motion artifact correction methods was evaluated with the following five metrics and compared with each method using ANOVA.<br><br>1. AUC0–2 seconds<br>2. AUC2–6 seconds<br>3. Ratio between AUC 2–6 and AUC 0–2<br>4. Within-subject variability<br>5. Number of Trials Retained<br><br>CBSI was effective in reducing noise quantitatively but sometimes produced inconsistent or unstable hemodynamic responses.	The tPCA is an effective and promising choice for correcting motion artifacts in fNIRS data from young children.	Run-time performance of the methods is not considered.<br><br>Newer methods (e.g., kurtosis-based wavelet) and hybrid artifact correction methods are not evaluated.

						PCA was found to be unviable for this dataset because it discarded too many trials.<br><br>Spline and tPCA were most effective for reducing within-subject variability and retained more trials.<br><br>Comparison between tPCA and Spline showed that tPCA consistently outperformed Spline.		
Hu et al. (2015) [63]	12 healthy children<br>8 females<br>age range 6.8 - 12.6<br>Mean age ± SD = 9.9 ± 1.75 years	Optical Density<br><br>10 Hz<br><br>fNIRS Wavelength: 690 nm and 830 nm	Motion artifacts were categorized into four types:<br><br>Type A: spikes with a standard deviation of 50 over the mean within one second (sudden move generated motion artifact)<br>Type B : Peaks with a standard deviation of 100	Left inferior frontal gyri (language-related area)	Spline<br><br>Wavelet<br><br>PCA<br><br>Moving average (MA)<br><br>CBSI<br><br>Wavelet + MA	Evaluated the effectiveness of different motion artifact detection and correction for four types of artifact categories.<br>t-values and R values of the GLM, AUC, and AUC ratio were used to evaluate these methods for each of the four different motion artifact types.	The Wavelet+MA combined approach demonstrated the best performance for reducing motion artifacts and achieved promising results across GLM metrics.	The Wavelet+MA combined approach demonstrated the best performance for reducing motion artifacts and achieved promising results across GLM metrics.<br><br>Generalizability of the used dataset, as it does not reflect the complexities of real-world fNIRS data.

			from the mean during a time portion ranging from 1 to 5 seconds<br>Type C : Gentle slope between 5 and 30 seconds with a standard deviation of 300 from the mean<br>Type D : Slow baseline shifting longer than 30 seconds with a standard deviation of 500 from the mean					

CBSI = Correlation-Based Signal Improvement, fNIRS = Functional Near-Infrared Spectroscopy, GLM = General Linear Model, HbO2 = Oxyhemoglobin, HbT = Total Hemoglobin, HHb = Deoxyhemoglobin, MA = Moving Average, PCA = Principal Component Analysis, SD = Standard Deviation, tPCA = Targeted Principal Component Analysis
